# Behavioral, Psychiatric, and Cognitive Adverse Events in Older Persons Treated with Glucocorticoids

**DOI:** 10.3390/medicines5030082

**Published:** 2018-08-01

**Authors:** Ciro Manzo, Jordi Serra-Mestres, Alberto Castagna, Marco Isetta

**Affiliations:** 1 Rheumatologic Outpatient Clinic and Geronthorheumatologic Service, 80065 Sant’Agnello, Italy; 2 Center for Cognitive Diseases and Dementias, 80038-ASL Napoli 3 Sud Pomigliano d’Arco, Italy; 3 Department of Old Age Psychiatry, Central and North West London NHS Foundation Trust, London UB8 3NN, UK; jordi.serra-mestres@nhs.net; 4 Center for cognitive diseases and dementias, Catanzaro lido, ASP Catanzaro, 88100 Catanzaro, Italy; albertocastagna@tiscali.it; 5 Library and Knowledge services, Central and North West London NHS Foundation Trust, London UB8 3NN, UK; marco.isetta@nhs.net

**Keywords:** glucocorticoids, elderly, behavioral adverse events, psychiatric adverse events, cognitive adverse events

## Abstract

**Background:** Since the introduction of glucocorticoids (GCs) in the physician’s pharmacological arsenal, it has been known that they are a cause of behavioral or psychiatric adverse events (BPAE), as well as of cognitive problems. To the best of our knowledge, the relationship between these adverse events and GCs in older persons has never been evaluated, except through case-reports or series with few cases. In this paper, a review of the literature regarding BPAEs and cognitive disorders in older people treated with CSs is undertaken. **Methods:** A comprehensive literature search for BPAEs was carried out on the three main bibliographic databases: EMBASE, MEDLINE and PsycINFO (NICE HDAS interface). Emtree terms were: Steroid, steroid therapy, mental disease, mania, delirium, agitation, depression, behavior change, dementia, major cognitive impairment, elderly. The search was restricted to all clinical studies and case reports with focus on the aged (65+ years) published in any language since 1998. **Results:** Data on the prevalence of the various BPAEs in older patients treated with GCs were very scarse, consisting mainly of case reports and of series with small numbers of patients. It was hence not possible to perform any statistical evaluation of the data (including meta-analysis). Amongst BPAEs, he possibility that delirium can be induced by GCs has been recently been questioned. Co-morbidities and polypharmacy were additional risk factors for BPAEs in older persons. **Conclusions:** Data on BPAEs in older persons treated with GCs, have several unmet needs that need to be further evaluated with appropriately designed studies.

## 1. Introduction

Glucocorticoids (GCs) are widely used medications and are highly effective in the management of a number of acute and chronic conditions, ranging from autoimmune and inflammatory disorders, to cancer, asthma, chronic pulmonary obstructive disease, pain, cerebral edema, etc. The systemic side-effects of these medicines (for example diabetes mellitus, osteoporosis, glaucoma, and cataracts) are well known to physicians [1].

In 1968, McEwen et al. demonstrated that in hippocampal neurons of adrenalectomized male rats, receptors with high affinity for ^3^H-corticosterone were present [2]. Now, we known that these receptors represent one of the two main types of GCs nuclear receptors, called mineralcorticoid receptors. They are mainly occupied by physiological levels of GCs. The glucocorticoid receptors represent the second type of GCs nuclear receptors and are meanly occupied when GC levels are increased, such as during therapy with GCs or during GC peak secretion in all stressful situations. Also, in the human brain, hippocampal CA1 neurons have the highest level of expression of GCs receptors [3,4]. These receptors are well expressed even in the elderly [5,6]. The mineralcorticoid receptors are mainly expressed in the hippocampus and the amygdala whereas the glucocorticoid ones are more widely expressed in stress-regulating centers such as the hippocampal-amygdala circuitry and the ascending aminergic neuronal networks. In other words, there is a balance between these two types of receptors, with their axis moving according to GCs levels. This balance is absolutely necessary for the anatomical-functional homeostasis of the brain (and of all other functions that depend on it). In the central nervous system (CNS), GCs can directly act on neurons, guiding their structural and functional changes, an expression of “neuronal plasticity”. This process is strictly linked to GCs dosage and their exposure time. So, acutely elevated GCs levels increase synaptic plasticity and facilitate hippocampal-dependent cognition while long-term exposure impairs synaptic plasticity and cognition, decreases neurogenesis and spine density, and causes dendritic atrophy. In addition to neurons, microglia cells also express GCr and play an important role in the GC-mediated damage to neurons. In fact, GCs can activate microglia cells, putting them in a “primed” or “sensitized” or “alert” state. As a consequence of this, microglia undergo changes such as cell surface up regulation of some receptors without production of mediators. Only in the presence of further stimuli, microglia begin to produce cytokines and other mediators of inflammation (tumor necrosis factor alfa and interleukin 1 beta, for example) at full strength, resulting in a neuronal damage. On the other hand, an over- or prolonged stimulation of microglia cells by GCs can lead to their activation and hypertrophy, resulting, for example, in neuropathic pain or an anxiety-like behavior. Ultimately, GC actions on neurons can be realized both directly and through microglial cells. Furthermore, these observations, emphasize that exposure to GCs can favor a pro-inflammatory response (instead of an anti-inflammatory action) in certain regions of the brain (hippocampus, cerebellum, pituitary–hypothalamus axis), if further stimuli are present [3].

Since the introduction of GCs in the physician’s pharmacological arsenal in the 1950s, it has been known that they are also a significant cause of behavioral or psychiatric adverse events (BPAE) [7], as well as of cognitive problems. Just in 1950, Boland and Headley noted that even after small doses of cortisone, almost every patient “experienced some psychic changes” [8]. BPAEs of GCs have been described both during the short-term administration as well as with their chronic use, and because of some present with a rapid and dramatic onset with psychotic and manic symptoms, they had originally been subsumed under the nosological rubric of “corticosteroid psychosis” [9]. However, there are psychiatric problems that arise in less dramatic way such as depression, anxiety, and cognitive problems.

Although age in itself has been reported to be unrelated to the incidence of BPAEs in those taking GCs [7,9], it has been suggested that the assessment of psychiatric symptoms in older persons requires a number of special considerations such as those relating to the presence of comorbid physical health disorders, the accrual of cognitive impairment, and even dementia, and the fact that older adults are more likely to be subject to polypharmacy, using drugs that in themselves may be associated with behavioral and psychiatric disturbances [9,10,11,12,13].

Nevertheless, to the best of our knowledge, the relationship between these adverse events and GCs in older persons has never been evaluated, except through case-reports or series with few cases. In this paper, a review of the literature of the last 20 years regarding BPAEs and cognitive disorders in older people treated with CSs is undertaken.

## 2. Methods

A comprehensive literature search was carried out on the three main bibliographic databases: EMBASE, MEDLINE and PsycINFO (NICE HDAS interface). The search strategies merged available subject headings and free text approaches to increase the yield for studies reporting on psychiatric sequelae of steroid administration. On EMBASE, for instance, Emtree terms-(exp STEROID/ or STEROID THERAPY/ and (exp *MENTAL DISEASE/ OR *MANIA/ OR *DELIRIUM/ OR *AGITATION/ OR exp *DEPRESSION/ OR *BEHAVIOR CHANGE/ OR DEMENTIAS/MAJOR COGNITIVE IMPAIRMENT)-were complemented by natural language-((steroid-induced).ti,ab AND ((psychos*).ti,ab OR (depress*).ti,ab OR (delir*).ti,ab OR (mania).ti, ab OR (“behav* change*”).ti,ab OR (psychiatr*).ti,ab) OR (dem*).ti,ab)). The search was restricted to all clinical studies and case reports with focus on the aged (65+ years) published in any language since 1998. Additional references were identified through other sources (i.e., cited by key reviews).

## 3. Findings

### 3.1. Risk Factors

It has been suggested that there are a number of factors that can be associated with the development of BPAEs as a result of treatment with GCs. They have been divided in patient, illness, and pharmacological factors [11].

#### 3.1.1. Patient Factors

Age has not been found to be important in determining the emergence of BPAEs, while gender was. A higher frequency of BPAEs in women has been almost constantly reported in the literature, with a 2/1 ratio. There are several hypotheses that have been formulated to explain these gender differences, but it may only be a consequence of a higher prevalence of diseases (such as Systemic Lupus Erythematosus, SLE or other autoimmune diseases) that requires GC treatment.

The presence of a previous psychiatric history cannot be a risk factor (and hence this should not be a contraindication to GC therapy [10]. However, the presence of previous GC-induced psychiatric adverse events may predispose to further episodes when re-challenged with these drugs, and a sensitization mechanism has been suggested [10].

#### 3.1.2. Illness Factors

It has been reported that suffering from an illness that is frequently associated with neuropsychiatric manifestations may increase the risk of GC-induced BPAEs [10]. This would refer to conditions such as SLE [14,15,16]. In addition, some cohort studies have indicated that the presence of hypoalbuminemia may also increase the risk of BPAEs from GCs, as lower levels of serum albumin would be related to higher levels of free and active GCs, which are normally inactive when bound to albumin [17].

#### 3.1.3. Pharmacological Factors

The most consistent finding relating to treatment since the very early reports is the effect of GC dose on the risk of developing BPAEs, with the risk increasing with higher GC doses [10,12]. Although most large studies are not necessarily focused on older adults, it is not clear whether in this group the risk is the same, bearing in mind what has been mentioned earlier in relation to the special considerations to be had in older people. Besides being linked to the systemic administration of GCs, BPAEs have also been reported in older adults receiving GCs via eye drops [18,19], intra-articular injections [17,20,21] and after a cervical epidural, four medial branch blocks, four trigger point injections, and a tendon injection in a shoulder, all including GCs [22]. It has been shown that methylprednisolone 80 mg injected into a knee joint with osteoarthritis leads to a mean plasma peak concentration of 169 ng/mL eight hours post-injection [23]. The mean concentration of GCs in plasma is also almost six times greater when two knees are injected compared to just one [24]. It has also been suggested that older adults are most at risk of systemic side-effects from eye drops [25], and that as much as 80% of the active principle of eye drops may enter the systemic circulation [26].

As previously mentioned, the dose of GCs is related to the risk of BPAEs, but there does not seem to exist a safe low dose of GCs [10,12].

BPAEs tend to arise within the first two weeks of treatment with GCs [9], but they can also develop after long periods of treatment [27], and even after discontinuation of long term GCs. In fact, it has been observed that patients on extended GCs therapy frequently develop withdrawal symptoms when treatment is stopped, suffering from symptoms such as fatigue, depression, discouragement, anorexia, or psychosis [27]. A cohort study (21,995 patients) of neuropsychiatric outcomes following discontinuation of long-term GCs (1–3 years) with a median age at first GCs course discontinuation of 72.6 years (interquartile range 61.3–80.5 years), found that discontinuation was associated with an increased risk of both depression and delirium, and that those treated with long-acting GCs were particularly at risk. In this cohort, older people were also at a higher risk of delirium [28].

Finally, and especially in older people in whom the likelihood of polypharmacy is higher, the role of concomitant pharmacological treatments needs to be taken into consideration [10]. Of specific importance are drugs that interact with the metabolism of GCs, especially those that can increase the plasma levels of unbound GCs such as clarithromycin [29].

### 3.2. Behavioural and Psychiatric Disorders Associated with CSs Use

#### 3.2.1. Delirium

Delirium is an acute disturbance in attention and awareness, tending to fluctuate in severity during the course of a day. A disturbance in cognition (e.g., memory deficit, disorientation, language, visuospatial ability, or perception) represents an additional classification criterion. According to the American Psychiatric Association (APA) Diagnostic and Statistical Manual of Mental Disorders (DSM), there must be evidence from the history, physical examination or laboratory findings that these disturbances are a direct consequence of another medical condition, substance intoxication or withdrawal (i.e., due to a drug of abuse or to a medication), or exposure to a toxin, or is due to multiple etiologies [30]. Delirium is associated with negative outcomes such as a short life expectancy, first of all. Furthermore, an increased risk of incident cognitive impairment and dementias has been observed in these patients. On the other hand, in some older persons, delirium can be superimposed on dementia [31,32]. There are three different types of delirium: (1) hyperactive (the most frequent); (2) hypoactive (more likely to go unrecognised; carphology is a characteristic sign); and (3) mixed.

The possibility that the onset of delirium is related to one or more precipitating factors, especially in older patients with multiple predisposing elements, has been frequently discussed in literature [33,34,35,36]. According to the theory proposed by Inouye et al, predisposing and precipitating factors are inversely proportional to the development of delirium.

We know that there is a relationship between GCs doses and the risk of developing acute adverse reactions. For example, in 1972, the Boston Collaborative Drug Surveillance Program published data on acute adverse reactions to prednisone in relation to dosage: Amongst 718 patients, only 1.3% developed psychosis with less than 40 mg prednisone/day compared to 18% taking more than 80 mg/day [12]. On the other hand, as highlighted above, it is widely accepted that the development of delirium originates from a complex inter-relationship between predisposing factors and exposure to noxious insults or precipitating factors. So, in clinical practice, the risk that low doses of GCs may induce a delirium in an elderly person with multi-morbidity, polypharmacy and a fragile homeostatic balance (“frail elderly”) must always be kept in mind. In the last few years, a new working-hypothesis was proposed. In “the systems integration failure hypothesis”, the specific cognitive and behavioral manifestations in patients with delirium are considered as the result of a combination of neurotransmitter function and availability, variability in integration and processing of sensory information, motor responses to both external and internal cues, and the degree of breakdown in neuronal network connectivity [37]. In this working-hypothesis, the role of GCs and their relationship with a disruption of circadian rhythms is speculatively interesting [38]. The potential consequences on this rhythm of the different formulation of prednisone, deserve to be investigated. In the last decade, the pharmaceutical industry has made available a modified-release prednisone for patients affected by rheumatoid arthritis to be taken at bedtime (around 22:00 hour) [39]. This formulation is more aligned with chronotherapy principles [39]. As for today, to the best of our knowledge, there is no data concerning the impact of traditional prednisone (taken on awakening) versus release-modified one as a precipitating factor for delirium. It has also been documented that previous psychiatric disorders in the patient’s medical history represent lesser risk factor for delirium than previous BPAEs [14]. However, two systematic reviews by the same investigators highlighted that the evidence about the possibility that GCs being associated with delirium must be evaluated as insufficient [40,41]. Lastly, there is scant mention of types of GCs-induced delirium (hyperactive, hypoactive, mixed) in the literature.

Key research gaps emerge on evaluation of the published literature: (1) Delirium is particularly common among hospitalized elderly people and the vast majority of data concerns this subset of patients. However, this population sample has different characteristics respect to the elderly living at home [42]. Data regarding delirium induced by GCs in elderly persons living at home is very scarce and not conclusive [43,44,45]; (2) As already highlighted, delirium can be superimposed on dementia or can represent the first clinical manifestation of dementias. In this case, do GCs induce delirium or dementia? This point is not trivial: in fact, dementia (with or without delirium) can be induced by GCs and delirium screening tools gave different results, depending on whether or not dementia was present [46,47]; (3) It may not be easy to navigate through the different, possible drug interactions in older patients with polypharmacy [48,49,50].

The above points can be considered as unmet needs to be evaluated with appropriate studies.

#### 3.2.2. Dementia

In Western countries, Alzheimer disease (AD) is the most frequent type of dementia. The hippocampus, which is affected in the early stages of classical or amnestic AD, has, as already highlighted, a high concentration of GCr; and therefore, plays an important role in cortisol regulation, inhibiting the hypothalamic-pituitary-adrenal axis after stressor-induced cortisol elevations, allowing cortisol increases after psychosocial stress, and controlling the cortisol awakening response [51]. So, every early pathology in the hippocampus before AD onset could alter cortisol regulation, resulting in unstable cortisol output and high cortisol variability [52]. Conversely, unstable cortisol fluctuations over time may also influence the health of the hippocampus [53,54]. Thus, the relationship between GCs and hippocampus is dynamically bi-directional. The effects of GCs on memory involve not only hippocampus but also other cerebral areas (pre-frontal cortex, above all) [55,56]. Therefore, it is not surprising that during therapy with GCs, modest and reversible deficits in memory (declarative memory, but also in attention and orientation) can be often observed [57].

In 1984, Varney et al. highlighted the possibility that a reversible dementia can occur during therapy with GCs without psychotic manifestations [58]. On the other hand, the presence of psychotic manifestations during therapy with GCs must be carefully evaluated in their relationship with a possible, concomitant dementia. [59,60]. In 2004 and then in 2007, Wolkowitz et al. published a case-series of patients with significant cognitive impairment during therapy with GCs. In these patients, this impairment persisted after GCs discontinuation as a consequence of steroid neuroendargement or neurotoxicity. They proposed the term of “steroid dementia syndrome” as the paradigm of a non-reversible dementia. [61,62].

The relationship between dementias and GCs must take into account also the disease for which GCs are used. In fact, some of these conditions can, in their selves, induce a cognitive impairment. An example of this is SLE [63,64].

The possibility that patients with dementia can favorably respond to GCs (rather than to other drugs such as acetylcholinesterase inhibitors, for example) must be considered in clinical practice. This possibility is self-evident for all the dementias consequence of a rheumatic autoimmune disease, such as SLE. More difficult is the diagnosis of the so-called “autoimmune encephalopathy”. In fact, this diagnosis is mostly taken into consideration only after other etiologies (structural, neoplastic or paraneoplastic, infectious, toxic, metabolic, vascular etiologies) have been excluded. In some cases, a pathogenic antibody specific (such as voltage-gated potassium channel antibodies or anti-NMDA receptor antibodies) are present; in other cases, is characteristic the association with Hashimoto’s thyroiditis (so-called “Hashimoto’s encephalopathy”) [65,66]. Lastly, in some patients, an autoimmune encephalopathy can suggest a primary central nervous system vasculitis [67].

High-dose corticosteroids (0.5–1.5 g. prednisone per day) are the initial treatment in most patients and this may serve as a diagnostic test when the diagnosis is uncertain. A complete (or near complete) resolution of cognitive impairment after a high dose GCs treatment is very suggestive. [68,69].

Exceptionally, has been documented a coincidence of AD or Lewy body disease with an autoimmune steroid-responsive encephalopathy [70]. In these patients, GCs treatment can realize a period (months or years) of improved cognitive impairment and quality of life. 

As for delirium, comorbidities and polypharmacy can be confounding factors in older persons with dementia.

#### 3.2.3. Mania and Psychosis

Under the rubric of “psychosis”, both acute affective and *schizophreniform* presentations have been described [11] which can be very disruptive and interfere with daily functioning, and will often require intervention [71]. In fact, affective disorders in the form of mania or hypomania are the most frequent BPAEs following the recent onset of treatment with GCs [72,73] with around 55% prevalence in one review [74]. A retrospective series of patients with more than one episode of GC-induced mood changes, found that 85% of the episodes were primarily manic in nature [75]. A milder form consisting of euphoria/elation with increased joviality and optimism can be seen more frequently in patients taking GCs [76,77]. Manic symptoms in older patients have also been reported to persist for many months after cessation of GCs [78], indicating that treatment may be needed for longer periods of time.

The symptoms of mania secondary to GCs are not different from those of primary mania, and the use of rating scales can help with the diagnosis; for example the Young Mania Rating Scale (YMRS) [79]. Mania secondary to GCs can also present with psychotic phenomena such as delusions and hallucinations, and with features suggestive of delirium. Mania can also be followed by depression, adopting a bipolar form.

A review of 53 cases, including patients over the age of 65, established that the frequency of psychosis was 62%, although this figure included psychoses in the context of mania, depression, and delirium, rather than paranoid or schizophreniform psychoses [74].

Paranoid psychoses can also occur although but their prevalence in older adults is not clear, as it is often difficult to distinguish them from manic presentations with psychotic symptoms. Psychotic phenomena can also occur in patients with delirium and hence a comprehensive assessment is required, especially in older people who are more at risk of delirium. Occasionally, catatonic states can also be precipitated by exposure to GCs [80].

#### 3.2.4. Depression

Depressive disorder is another highly frequent BPAE in patients on GCs, with a prevalence of 24% in one review [74]. A very large cross-sectional community study in Canada (Canadian Health Survey) found that GC-treated individuals had a statistically significant increase in the prevalence of depression compared to those not treated with GCs [81], with an estimated 1 year prevalence three times higher than in non-treated subjects, irrespective of age, gender, or perceived health. In neurons with glucocorticoid receptors, the steroid-receptor complex goes into the nucleus and alters gene transcription, resulting in alterations in the production of neurotransmitters (including dopamine and serotonin) and neuropeptides (e.g., somatostatin, beta-endorphin). Concentrations of serotonin receptors at the synaptic cleft are also affected, and may contribute to mood symptoms in corticosteroid-treated patients [82].

Depression can occur shortly after initiation of treatment with GCs and in those on long-term GCs. It can also occur in the context of a GC-withdrawal syndrome, especially after long-term use. It has been suggested that the risk of depression in GC-treated patients increases with prolonged or chronic exposure [74].

Self-harm and suicidality have been reported in GC-treated patients, often in the course of depression [75], and in one review including older adults, the frequency of suicidal ideation was 36% [74]. Catatonia, in the form of depressive stupor, can also complicate depressive episodes [75].

The use of rating scales in non-psychiatric settings could help to identify depression in older patients taking GCs or having recently come off them (e.g., The 15-item Geriatric Depression Scale (GDS), or the Hospital Anxiety and Depression Scale (HADS) [83,84].

#### 3.2.5. Anxiety and Panic

Anxiety disorders have been reported in patients treated with GCs, adopting the profiles of generalized anxiety, a free floating form of anxiety, as well as of panic disorder. The literature is very scarce on anxiety disorders in this group, and especially in older adults.

### 3.3. Treatment Considerations

The literature on treatment of GC-induced BPAEs is limited to case series, case reports, and small trials, and data specific to older patients is scant. Large RCTs are needed to establish a more robust evidence-base. It is also very important to raise awareness of the risk of BPAEs with GC treatment amongst clinicians as well as among patients and their families and careers, so that early diagnosis and intervention can take place [74]. Prior to starting treatment with GCs, it is imperative to review the patient’s current medication list to ensure that is rationalized if possible, and that any drugs that may interact with GCs are taken into account. The background medical condition that is being treated with GCs needs to be considered too as some illnesses may be associated with neuropsychiatric symptoms in any case (for example, SLE).

Once a BPAE has been identified, the first step in its management should be the reduction of the dose of GC or its tapering off if at all possible. This can result in the resolution of symptoms [9,10,14,60,61,77].

In relation to the treatment of manic episodes, an open label trial found that olanzapine was efficacious in GC-induced mania and mixed mania [85]. Lithium, sodium valproate, carbamazepine, risperidone, quetiapine, haloperidol, lamotrigine, gabapentin, and clonazepam, have all been reported to have been helpful in the treatment of GC-induced mania [10,14,72,74]. These drugs can also be effective even in the treatment of GC-induced delirium and of GC-induced behavioral and psychological symptoms during dementia (BPSD) [86].

Little evidence exists on how to treat or to prevent cognitive changes associated with use of exogenous corticosteroids. In a randomized, double-blind, placebo controlled study, Brown et al. hypothesized that giving phenytoin to patients receiving oral corticosteroids would improve declarative memory. The results failed to show a statistically significant difference in declarative memory between controls and patients receiving phenytoin; however, they found that phenytoin reduced the incidence of hypomania associated with corticosteroids. Possibly, this is related to the fact that phenytoin interferes with cytochrome P450 activity, increasing GCs hepatic metabolism and thereby accelerates their clearance [87].

In a randomized placebo-controlled double-blind study, administration of 20 mg/day of memantine to patients receiving prednisone produced an improvement in memory compared with controls on an equivalent dose of prednisone while receiving placebo [88].

In the case of older adults, a number of considerations are required when choosing a pharmacological intervention, such as the potential toxicity of lithium with worsening renal function in addition to its own potential for causing renal impairment, the increase in the risk of falls with antipsychotics, valproate, and benzodiazepines, the increased risk of extrapyramidal side-effects from antipsychotics, and the increased risk of cardio-metabolic disorders with atypical antipsychotics in this age group [89].

GC-induced depression has been reported to have responded to lithium [90], as well as to lithium in combination with antidepressants, to tricyclic antidepressants, selective serotonin reuptake inhibitors (including fluoxetine, sertraline, and fluvoxamine), and venlafaxine [74]. Electroconvulsive Therapy has been beneficial in severe cases and in psychotic depressive episodes [91]. In older adults it is probably less advisable to use tricyclic antidepressants due to their strong anticholinergic effects which may worsen or precipitated delirium, in addition to other substantial side-effects.

As reported above, a significant proportion of patients receiving GC treatment may develop suicidal ideation, and clinicians should be mindful of this fact and inform patients and families to be vigilant in this regard.

GC-induced psychosis has been reported to respond to both typical and atypical antipsychotics [10].

## 4. Conclusions

Even if widely used, the available literature on the prevalence of the various BPAEs in patients treated with GCs is not very extensive, consisting mainly of case reports and of series with small numbers of patients [92].

As highlighted in our review article, the literature on the same issue specifically concerning older patients is even scarcer. This makes it no possible to carry out any statistical evaluation of the data (including meta-analysis). Furthermore, in older patients, polypharmacy, multimorbidities and frailty (usually absent in other age groups) can be confounding factors and represent critical issues in the treatment of BPAEs. Lastly, diverse affective, behavioral, and cognitive syndromes have often been lumped together under the term *steroid psychosis* (whether or not a patient has been psychotic). The use of the same label for myriad syndromes limits our ability to establish their etiology, treatment, and outcome with more certainty and confidence [75]. More appropriately designed studies in the age group are needed to improve our knowledge of these BPAEs. As highlighted by some investigators [11,73], clinical reports are necessary for hypothesis making whereas clinical trials are necessary for hypothesis testing. These trials should include, as above underlined, formal psychiatric assessment; consider all the possible confounding factors; present more information on long-term outcome and on the risk of BPAEs recurrence; have data regarding the impact of the duration of GC treatment on the onset of BPAEs in the age group (data that are currently lacking). In the next studies in older persons, these needs should be properly satisfied.
